# Formulation and Characterization of Oral Mucoadhesive Chlorhexidine Tablets Using *Cordia myxa* Mucilage

**Published:** 2012-10-07

**Authors:** Eskandar Moghimipour, Nasrin Aghel, Akram Adelpour

**Affiliations:** 1 Medicinal Plant Research Center, Faculty of Pharmacy, Ahvaz Jundishapur University of Medical Sciences, Ahvaz, IR Iran; 2 Cellular and Molecular Research Center, Ahvaz Jundishapur University of Medical Sciences, Ahvaz, IR Iran

**Keywords:** Mucoadhesive tablets, *Cordia myxa* mucilage, HPMC

## Abstract

**Background:**

The dilution and rapid elimination of topically applied drugs due to the flushing action of saliva is a major difficulty in the effort to eradicate infections of oral cavity. Utilization a proper delivery system for incorporation of drugs has a major impact on drug delivery and such a system should be formulated for prolonged drug retention in oral cavity.

**Objectives:**

The aim of the present study was the use of mucilage of *Cordia myxa* as a mucoadhesive material in production of chlorhexidine buccal tablets and its substitution for synthetic polymers such as HPMC.

**Materials and Methods:**

The influence of mucilage concentration on the physicochemical responses (hardness, friability, disintegration time, dissolution, swelling, and muco-adhesiveness strength) was studied and swelling of mucilage and HPMC were compared. The evaluated responses included pharmacopoeial characteristics of tablets, the force needed to separate tablets from mucosa, and the amount of water absorbed by tablets.

**Results:**

In comparison to HPMC, the rise of mucilage concentration in the formulations increased disintegration time, drug dissolution rate, and reduced MDT. Also, compared to 30% HPMC, muco-adhesiveness strength of buccal tablets containing 20% mucilage was significantly higher.

**Conclusions:**

It can be concluded that the presence of *Cordia myxa* powdered mucilage may significantly affect the tablet characteristics, and increasing in muco-adhesiveness may be achieved by using 20% w/w mucilage.

## 1. Background

The dilution and rapid elimination of topically applied drugs due to the flushing action of saliva is a major difficulty in the effort to eradicate infections of the oral cavity. Utilization a proper delivery system for incorporation of drugs has a major impact on drug delivery and such a system should be formulated for prolonged drug retention in oral cavity. Efficient local delivery of such as dental bleaches and antimicrobials to oral cavity is compromised by a number of factors that dramatically reduce residence time, most notably the shear forces associated with speaking, swallowing and mastication, as well as dilution and washout caused by continuous saliva production (1).

Polyacrylic acid, polymethacrylate acid, cellulose derivatives, polyethylene oxide, lectin, and chitosan are among polymers that have been used as mucoadhesive drug carriers (2). By actives using of copolymers, polymer conjugates, and interpolymer complexes, the mucoadhesive properties of polymers have been improved. Buccal patches are generally based on bio-adhesive polymers which, once hydrated, adhere to the buccal mucosa and withstand salivation, tongue movements, and swallowing for a significant period of time. The following characteristics are the basic requirements needed for a successful buccal delivery: a bio-adhesive to retain the drug in oral cavity and maximize the intimacy of contact with mucosa, a vehicle that releases the drugs at an appropriate rate under the conditions prevailing in the mouth, and strategies for overcoming the low permeability of the oral mucosa. The bio-adhesive bonds are formed by three steps: wetting and swelling of mucoadhesive polymer, entanglement of polymer and mucin chains, and formation of weak chemical bonds between entangled chains and polymer (3).

Chlorhexidine is a bis-biguanidine used to treat skin and mucosa infections. Its effectiveness against a wide range of microbial species has been previously proved. Using chitosan films, the release of chlorhexidine from the hydrogels has been maintained for 3 h (4). Also, it has been demonstrated that the topical use of bio-adhesive chlorhexidine gel during the postoperative week may decrease the risk of alveolar osteitis (5). Mucous membranes are moist surfaces lining the walls of various body cavities including gastrointestinal and respiratory tracts. They consist of a connective tissue layer (the lamina propria) above which is an epithelial layer, the surface of which is made moist usually by the presence of a mucus layer. Mucus is present in two forms: as a gel layer adherent to the mucosal surface, and as a luminal soluble or suspended form. The major components of all mucus gels are mucin glycoproteins, lipids, inorganic salts, and water; the latter accounts for more than 95% of gel weight, making it a highly hydrated system (6). The gel-like characteristic of mucin is due to glycoproteins that are the most important structure-forming components of mucin. Thickness of mucus layer is ranging from 50-450 μm (7, 8) to less than 1 μm, depending on the type of mucosal surfaces (9). Protection and lubrication are the major mucus functions.

The fruit of *Cordia myxa* has been used as an astringent, anthelminthic, diuretic, and demulcent agent as well as for the treatment of urinary tract infections, and diseases of the lung and spleen. Although the fruits known as bamber have been traditionally used for the treatment of lung infections and asthma, it has not been investigated scientifically. The effects of plant fruits on dilatation of peripheral blood vessels and contractility of ileum have been investigated (10).

## 2. Objectives

In the present study, using *Cordia myxa* mucilage, tablets containing chlorhexidine were formulated and evaluated for their releasing behavior and bio-adhesiveness strength.

## 3. Materials and Methods

### 3.1. Materials

Chlorhexidine was purchased from Shahre Daru, Tehran, Iran. Hydroxypropyl methylcelluose (HPMC), lactose, and magnesium stearate were obtained from Merck (Germany). Avicel from FMC Biopolymer (Ireland) was also used. Other materials and solvents were of analytical grade.

### 3.2. Extraction of Mucilage

1 Kg from fruits of *Cordia myxa* were soaked in double distilled water and boiled for 5h in a water bath until slurry was formed. The slurry was cooled and kept in refrigerator overnight so that most of its non-dissolved portion was settled out. The upper clear solution was decanted off. The supernatant was concentrated at 60°C on a water bath until the volume reduced to one third of its original volume. Solution was cooled down to the room temperature. The precipitate was dried under vacuum. The dried material was powdered and kept in a desiccator (11).

### 3.3. Study of Swelling Properties of Mucilage and HPMC

Mucilage and HPMC, 1 mg of each, were allowed to hydrate separately in 25 ml of distilled water at 25°C in a 25 ml graduated cylinder. The volumes of mucilage and HPMC were measured at 5min intervals until complete hydration (11).

### 3.4. Preparation of Bio-adhesive Tablets

Chlorhexidine buccal adhesive tablets were prepared by direct constant compression pressure using mixtures of the ingredients. The composition of the tablet formulations is given in Table 1. In addition to mentioned ingredients (Table 1), all formulations were contained 2% chlorhexidine, 10% Avicel, and 1% magnesium stearate as lubricant.

**Table 1:** Composition of Chlorhexidine (55.5 mg) Mucoadhesive Tablet Formulations

Formulation	HPMC, %	Powdered *Cordia myxa* Mucilage, %	Lactose, %
F_1_	30	-	57
F_2_	-	5	82
F_3_	-	10	77
F_4_	-	20	67

All formulations were contained chlorhexidine (2%), Avicel (10%), and Magnesium stearate (1%)

### 3.5. Hardness and Friability

The resistance of tablets against shipping or breaking under the condition of storage, transportation, and handling before administration depends on its hardness. The hardness of tablets of each batch was measured by Dr. Schleuniger pharmaton hardness tester. The hardness was measured in terms of Kg. The friability of tablets was determined using ERWEKA friabilator. 

### 3.6. Content Uniformity

For content uniformity test, representative samples of 30 tablets were divided into 3 groups (10 tablets in each) and assayed by spectrophotometer (Biochrome, WPA Biowave II, UK) at 257nm, individually. According to the standards, the measured value of at least 9 tablets must be within ± 15% of the declared potency and none may exceed ± 25%.

### 3.7. Disintegration

The disintegration test was carried out using a disintegration tester consisting of a basket rack holding 6 plastic tubes, open at the top and bottom, and the bottom of each was covered by a 10-mesh screen. The basket was immersed in a bath of suitable liquid held at 37 °C, preferably in a 1L beaker. For compressed uncoated tablets, the testing fluid was water at 37 °C. The test was repeated using 6 tablets.

### 3.8. Dissolution

The dissolution test was performed using a dissolution test apparatus (Erweka, DT 800, Germany) rotating at 100 rpm, as instructed by USP 24 for paddle method (6). The dissolution medium was 900 ml phosphate buffer (pH 6.8) and the temperature was set at 37 °C. Samples of the solution were withdrawn at definite time intervals. The dissolution media was then replaced by fresh dissolution fluid to maintain a constant volume. The solution was passed through a filter after which the chlorhexidine concentration was measured by an ultraviolet spectrophotometer (Biochrom, England) at a wavelength of 257 nm. The test was carried out in triplicate and the results were expressed as mean dissolution time (MDT) ± standard deviation (SD) (12).

### 3.9. Study of Tablets Swelling 

Three buccal tablets were weighed individually (W_1_) and placed separately in 2% agar gel plates with the core facing the gel surface, and incubated at 370C ± 10C. At regular 1 h time intervals during a 6 h period, the tablets were removed from the Petri dish and excess surface water was removed carefully with filter paper. The swollen tablets were then reweighed (W2) and the swelling indices (SI) were calculated using the formula given in the following equation (13).

Swelling Index = [(W_2_-W_1_)/ W_1_] × 100

### 3.10. Ex-vivo Mucoadhesive Strength Determination

Muco-adhesion evaluation of different formulations was carried out using texture analyzer and cow gut mucosa. Freshly excised cow gut mucosa was obtained from the local slaughter house. The tissue was placed in phosphate buffer (pH = 6.8), then cut in small pieces and placed on a holder using clips. The prepared tablets were attached to the probe using double sided tapes. The probe was lowered at a speed of 5 mm/min until the tablet made contact with the surface of mucosal tissue. A constant force of 10 N was applied for 5min, after which the probe was withdrawn at a speed of 0.5 mm/min. Peak detachment force was used to establish mucoadhesive strength using texture exponent software. The test was carried out for 10 tablets of each formulation (14).

### 3.11. Statistical Analysis

Tukey's post-hoc analysis was utilized to compare the dissolution and bio-adhesion data. Microsoft Excel Software was used to plot the results.

## 4. Results

**Figure 1:**
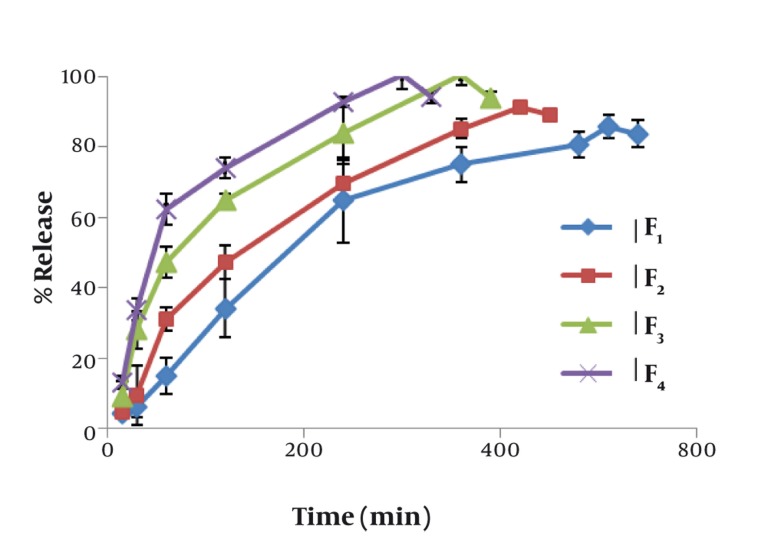
In Vitro Release of Chlorhexidine from Formulations F_1_ (30% HPMC), F_2_ (5% mucilage), F_3_ (10% mucilage), and F_4_ (20% mucilage)

Figure 1 shows the effect of concentration of mucilage on the release rate of chlorhexidine. The lowest release rate was observed with formulation F_1_ containing 30% HPMC. The release rate increased significantly (P < 0.01) by increasing the concentration of mucilage. The mucoadhesive strengths of mucoadhesive tablets are shown in Table 2. The results show that mucoadhesive strength increased in proportion with the increase of mucilage concentration. On the other hand, the lowest mucoadhesive strength was manifested by formulation F_2_ which contained 5% mucilage. Formulation F_4_ had the highest mucoadhesive strength that was significantly more than that of HPMC containing formulation (P < 0.01).

**Table 2:** Mucoadhesive Strengths, MDT, Disintegration time, and Hardness of Chlorhexidine Tablets made of HPMC and Different concentrations of Dried *Cordia myxa* Mucilage

Formulation	The Force Needed for Separation, No.	Mean Dissolution Time, min	Mean Disintegration Time, min	Hardness, Kg
F_1_	22.1 ± 1.197	184.37 ± 29.21	20.0 ± 4.0	4.6 ± 1.2
F_2_	8.2 ± 1.229	149.84 ± 19.83	14.5 ± 1.0	5.1 ± 1.5
F_3_	13.1 ± 1.852	99.78 ± 12.51	12.0 ± 1.0	5.6 ± 1.0
F_4_	26.2 ± 1.873	77.85 ± 7.15	9.0 ± 0.5	5.5 ± 1.2

## 5. Discussion

Among the various transmucosal routes, buccal mucosa has excellent accessibility, an expanse of smooth muscle, and relatively immobile mucosa, hence suitable for administration of retentive dosage forms. Microparticulate bio-adhesive systems are particularly interesting as they offer protection to therapeutic entities as well as enhanced absorption that result from increased contact period provided by bio-adhesive ingredients (15). Several synthetic and semi-synthetic compounds have been previously utilized to exert muco-adhesiveness, although there are few reports in utilization of pure herbal products. Combination of HPMC and pectin has been used to retard drug release from diltiazem sublingual tablets. The prepared tablets were of satisfactory hardness and showed good adhesion to rat bio-membrane (16). Our results indicated that incorporation of HPMC in chlorhexidine tablets decreased the releasing rate of drug, probably because of absorption of water by the polymer and forming a gelatinous barrier layer at the surface of the tablet matrix, while the mucilage of *Cordia myxa* did not form a gel layer and therefore did not show any significant decrease in drug release. Also, the increase in mucilage level in the tablet resulted in increased penetration of solvent molecules into the matrix and outward diffusion of drug molecules into the dissolution medium. The results of our study showed the significant effectiveness of *Cordia myxa* mucilage on drug release profile. Compared to HPMC containing tablets, the mucilage containing formulations showed faster drug release, and also the maximum release have been raised. Moreover, the impact of mucilage concentration was significant. It has been previously shown that drug release and bio-adhesion properties of buccal tablets can be controlled by changing polymer type and concentration. Bio-adhesion of the developed formulations provided a longer residence time, thus reducing loss of drug by swallowing, that may result in improved bioavailability (17). Water diffusivity usually depends on the total concentration of viscosity-inducing agents in a system that governs water diffusion into matrix systems. Furthermore, erosion can play a role in drug release. Water soluble drugs are released primarily by diffusion of dissolved drug molecules across the gel layer whilst poorly water soluble drugs are released predominately by erosion mechanisms (18). Drug release from swollen polymer matrices is based on glassy-rubbery transition of the polymer which occurs as a result of water penetration into the matrix. Although interactions between water, polymer, and drug are the primary factors in release control, several formulation variables also influence drug release rate to greater or lesser degrees (19).

In the buccal region, a tablet may be adhered either to buccal (cheek) or gingival tissues. For local drug delivery, the highly keratinized epidermis of the gingival tissue will present a barrier to systemic absorption. Examples of oral cavity diseases for which buccal dosage forms have been designed include aphthous stomatitis, oral candidiasis, and periodontal disease (20). Our results showed that the muco-adhesion strengths of F_2_ and F_3_ which contained lower concentrations of mucilage were significantly less than that for HPMC based formulations. By increasing the amount of mucilage up to 20% in F_4_, the mucoadhesive strength was increased significantly (P < 0.01). The comparison between adhesion results of F_1_ and F_4_ showed the higher efficiency of mucilage as mucoadhesive agent. The results of a study showed that muco-adhesion strength was significantly increased by increasing the contact time. This was consistent with those obtained by Tobyn et al. and Wong et al. in which different types of polymers (e.g. carbomer, polycarbophil, HPMC, NaCMC) and model mucosa were used. Increasing contact time may provide interdiffusion and chain entanglement between polymer and mucin chain in mucus membrane. An increase in contact resulted in an increase in formation of secondary bonds and diffusion path or depth of interpenetration between two macromolecules. Hence, contact time is important to allow sufficient hydration, swelling, interpenetration, and bond formation for muco-adhesion (21). Also Leung and Robinson demonstrated that muco-adhesion of carbomer was a time-dependent process supporting the proposed interpenetration as being a time-dependent process (22). In a study, increasing contact time between mucoadhesive polymer and mucus layer leaded to increase the mucoadhesive strength (18). Nevertheless, Wong et al. observed that no significant increase in the mucoadhesive strength was seen at a contact force above 0.5 N, due to a maximum intimate contact. They suggested that too high contact force may not be advantageous but may damage the mucosa without achieving better result (23). Although, it has been reported that preparations with separate adhesive and drug release parts may provide a more satisfactory bioavailability (24).

It can be concluded that increasing of tablet hardness, shortening of disintegration and dissolution times, and increasing of muco-adhesiveness may be achieved by using 20% w/w *Cordia myxa* mucilage. Also due to the herbal nature and high biocompatibility of the mucilage, it is suggested that it could be considered as alternative candidate for synthetic polymers in the formulation of buccal mucoadhesive tablets.
